# Cerebral venous sinus thrombosis and exploration of prognostic factors: a case series

**DOI:** 10.3389/fneur.2024.1359786

**Published:** 2024-11-14

**Authors:** Jiahui Liu, Junfeng Yang, Yu Fan, Changchun Jiang

**Affiliations:** Department of Neurology, Baotou Central Hospital, Baotou, China

**Keywords:** cerebral venous sinus thrombosis, etiology, radiological location, treatment, prognosis

## Abstract

**Background:**

This study examined the etiology, treatment response, and prognosis of patients with cerebral venous sinus thrombosis (CVST) at a single institution.

**Methods:**

This retrospective study included patients diagnosed with CVST between January 2016 and December 2020 at Baotou Central Hospital. The data were collected from patient charts, including sex, age, comorbidities, pregnancy, smoking, alcohol drinking, symptoms at onset, radiological location, examinations, Glasgow coma scale (GCS) at admission, National Institutes of Health stroke scale (NIHSS) at admission, modified Rankin scale (mRS) at admission, and treatments.

**Results:**

The study included 31 patients (13 males and 18 females) aged 39.0 (interquartile range, 30.0–53.0) years. Three (9.7%) patients had a history of hypertension, one (3.2%) had a history of stroke, four (12.9%) had thrombotic disorders, six (19.4%) were pregnant (including three who delivered), four (12.9%) were smoking, and four (12.9%) were drinking alcohol. The GCS at admission was 15.0 (IQR, 15.0–15.0), the NIHSS at admission was 0.0 (IQR, 0.0–2.0), and the mRS at admission was 0.0 (IQR, 0.0–0.0). The patients were grouped according to their mRS (>0 vs. 0); there were no significant differences between the two groups regarding the patient characteristics (all *p* > 0.05). Only an NIHSS of >0 at follow-up was associated with a 3-month mRS >0 (*p* < 0.001).

**Conclusion:**

The median age of the patients with CVST was 39 years. The majority were female (58%), 13% had thrombotic disorders, and 19% were or were recently pregnant. Only a NIHSS of >0 at follow-up was associated with a 3-month mRS >0.

## 1 Introduction

Cerebral venous sinus thrombosis (CVST) is a rare form of stroke and refers to thrombosis of the cerebral sinuses and/or cerebral veins (1). CVST generally occurs in young patients (typically <50 years old) (1, 2) and affects women more than men (2). CVST accounts for 0.5–1% of all strokes (1), with an incidence of about 5–16 cases per million people annually (1, 2). The presentation is highly variable, and symptoms can take a few weeks to develop (3, 4). The common risk factors for CVST include high-risk thrombophilia (e.g., antithrombin III, protein C and protein S deficiency, homozygosity for either factor V Leiden, or prothrombin G20210A mutations), use of oral contraceptives, pregnancy, malignancy, infection, trauma, and populations in developing countries (1, 2, 5). The complications of CVST include intracranial hemorrhage, intracranial hypertension, and seizures (1). Mortality is 4–5% in the acute phase (2).

The risk factors for 30-day mortality include depressed consciousness, altered mental status, thrombosis of the deep venous system, right hemisphere hemorrhage, and posterior fossa lesions (1, 6, 7). The factors associated with poor short-and long-term outcomes include demographic factors (age > 33–37 years and male sex), clinical factors (coma, neurologic deficit on National Institutes of Health Stroke Scale (NIHSS), encephalopathy, decreased level of consciousness, hemiparesis, and seizures), neuroimaging factors (intracerebral hemorrhage, involvement of straight sinus, thrombosis of deep vein system, and venous infarction), and other risk factors (cancer, central nervous system infection, and underlying coagulopathy hereditary thrombophilia) (1, 8).

Nevertheless, the prognostic factors of CVST remain poorly understood, mainly because of the small number of cases. Furthermore, a study revealed that the epidemiology of CVST significantly changed from 1942 to 2012 because of improvements in treatments, changes in risk factors, and improvements in diagnostic modalities leading to mild cases being diagnosed (9). The exact natural history and prognosis of patients with mild CVST are poorly known since they were not included in early studies. A study tried to develop a risk score to predict the outcomes of CVST, but its predictive value was low (10). Therefore, recent data on the prognosis of CVST are necessary. Evidence regarding the etiology, treatment response, and prognosis of CVST is still lacking.

Therefore, this study aimed to examine the etiology, treatment response, and prognosis of patients with CVST at a single institution. The results could help improve the management of patients with CVST.

## 2 Methods

### 2.1 Study design

This retrospective study included patients diagnosed with CVST between January 2016 and December 2020 at Baotou Central Hospital. The study was approved by the ethics committee of Baotou Central Hospital (approval No. 2023-WZ-081). The requirement for individual informed consent was waived by the committee because of the retrospective nature of the study. The study followed the principles of the Declaration of Helsinki and its amendments.

### 2.2 Population selection

The inclusion criteria were (1) diagnosed with CVST and (2) complete dataset. The diagnosis of CVST was based on the detection of sinus abnormalities using MRV and/or DSA in combination with clinical symptoms (11). The exclusion criteria were (1) minors, (2) patients with a Glasgow Coma Scale (GCS) score of 3–5 at admission, cerebral hernia at admission or during hospitalization, or death at any time, or (3) liver or kidney function serious failure, or coagulation function serious disorders.

### 2.3 Variable definition

The variables included sex, age, comorbidities (hypertension, diabetes, coronary artery disease, hyperlipidemia, history of stroke, and thrombotic disorders), history of pregnancy, history of delivery, smoking, alcohol drinking, symptoms of onset (headaches, limb numbness, speech impairment consciousness disturbances, limb tremors/convulsions, vomiting, memory impairment, seizures, and visual abnormalities), radiological location (straight sinus, transverse sinus, sigmoid sinus, superior sagittal sinus, internal jugular vein, and cortical veins, laterality, and venous involvement), examinations (magnetic resonance venography (MRV) and digital subtraction angiography (DSA)), Glasgow coma scale (GCS) at admission, NIHSS at admission, modified Rankin scale (mRS) at admission, anticoagulation treatment treatments, hemorrhagic transformation, drainage obstruction, venous recanalization, intracranial pressure, onset to admission time, and stage (acute, subacute, or chronic). Hemorrhagic transformation was defined as hemorrhagic infarction that occurred after venous thrombosis (12). Drainage obstruction was defined as obstruction of venous drainage resulting in swelling or hemorrhage in the brain (1). Venous recanalization was defined as the successful management of thrombosis and restoration of blood flow (13). The acute stage was <48 h from onset, the subacute stage was 48 h to 30 days after onset, and the chronic stage was >30 days after onset (14).

### 2.4 Data collection

All data were collected from the patient charts. As a retrospective study, the follow-up was solely based on the patient charts and was limited to the 3-month follow-up visit. Only 22 patients had symptoms described. The remaining nine patients were considered as having no symptoms.

### 2.5 Outcome

The patients were grouped according to the mRS (>0 vs. 0). The outcome was the mRS at 3 months.

### 2.6 Statistical analysis

All statistical analyses were conducted using R version 4.3.1. Descriptive statistics were performed to summarize the baseline characteristics of the patients. The Shapiro–Wilk test was employed to assess normality for continuous data. Data following the normal distribution were described using means ± standard deviations and analyzed using Student’s t-test; otherwise, medians and interquartile ranges (IQRs) were used for description, and the Mann–Whitney U-test was used for analysis. Categorical variables were described using frequencies and percentages and analyzed using the chi-squared test or Fisher’s exact test. Two-tailed *p*-values <0.05 were considered statistically significant.

## 3 Results

### 3.1 Characteristics of the patients

This study included 31 patients (13 males and 18 females) aged 39.0 (IQR, 30.0–53.0) years. No patients met an exclusion criterion. Three (9.7%) patients had a history of hypertension, one (3.2%) had a history of stroke, four (12.9%) had thrombotic disorders, six (19.4%) had history of pregnant (including three who delivered), four (12.9%) were smoking, and four (12.9%) were drinking alcohol (Table 1).

**Table 1:** Characteristics of the patients.

	*n* = 31
Sex	
Male	13 (41.94%)
Female	18 (58.06%)
Age (years)	39 (30, 53)
Hypertension	3 (9.68%)
Diabetes	0
Coronary artery disease	0
Hyperlipidemia	0
History of stroke	1 (3.23%)
Thrombotic disorders	4 (12.90%)
History of pregnant	6 (19.35%)
History of delivery	3 (9.68%)
Smoking	4 (12.90%)
Alcohol drinking	4 (12.90%)
Symptoms at onset	
Headaches	22 (70.97%)
Limb numbness	6 (19.35%)
Speech impairment	4 (12.90%)
Consciousness disturbances	2 (6.45%)
Limb tremors/convulsions	2 (6.45%)
Vomiting	2 (6.45%)
Memory impairment	2 (6.45%)
Seizures	2 (6.45%)
Visual abnormalities	1 (3.23%)
Radiological location	
Straight sinus	10 (32.26%)
Transverse sinus	22 (70.97%)
Side	
Not specified	3 (13.64%)
Left	9 (40.91%)
Right	9 (40.91%)
Bilateral	1 (4.55%)
Sigmoid sinus	14 (45.16%)
Side	
Not specified	10 (71.43%)
Left	1 (7.14%)
Right	2 (14.29%)
Bilateral	1 (7.14%)
Superior sagittal sinus	15 (48.39%)
Side	
Left	15 (100.00%)
Internal jugular vein	2 (6.45%)
Side	
Not specified	1 (50.0%)
Left	1 (50.0%)
Cortical veins	1 (3.23%)
MRV was performed	25 (80.65%)
DSA was performed	27 (87.10%)
GCS at admission	15 (15, 15)
NIHSS at admission	
0	18 (58.06%)
1–4	6 (19.35%)
≥5	7 (22.58%)
mRS at admission	
0	25 (19.35%)
>0	6 (80.65%)
Anticoagulation treatment	
300,000 U of urokinase in venous sinus in the morning and evening for 3 days	24 (77.42%)
500,000 U of urokinase in venous sinus once	1 (3.23%)
Balloon dilatation	2 (6.45%)
Warfarin 3 mg orally once daily for 3 months	4 (12.90%)
Hemorrhagic transformation	1 (3.23%)
Drainage obstruction	28 (90.32%)
Venous recanalization	30 (96.77%)
Intracranial pressure (mmH_2_O)	230 (210, 310)
Onset to admission time (days)	5 (3, 10)
Venous involvement	3 (9.68%)
Stage	
Acute	22 (70.97%)
Subacute	7 (22.58%)
Chronic	2 (6.45%)

MRV, magnetic resonance venography; DSA, digital subtraction angiography; GCS, Glasgow coma scale; NIHSS, National Institutes of Health stroke scale; mRS, modified Rankin scale.

Headaches were observed in 22 patients (70.97%), limb numbness in six (19.35%), speech impairment in four (12.9%), consciousness disturbances in two (6.45%), limb tremors or convulsions in two (6.45%), vomiting in two (6.45%), memory impairment in two (6.45%), seizures in two (6.45%), and visual anomalies in one (3.23%) (Table 1).

Twenty-five (80.6%) patients underwent MRV, and 27 (87.1%) underwent DSA. The lesions were in the straight sinus in 10 (32.3%) patients, transverse sinus in 22 (71.0%), sigmoid sinus in 14 (45.2%), superior sagittal sinus in 15 (48.4%), internal jugular vein in two (6.45%), and cortical veins in one (3.23%) (Table 1). Three patients had venous involvement: the cortical vein in one patient, the right internal jugular vein in one, and the left internal jugular vein in one. For the other patients, only venous sinuses were involved.

The GCS at admission was 15.0 (IQR, 15.0–15.0), the NIHSS at admission was 0.0 (IQR, 0.0–2.0), and the mRS at admission was 0.0 (IQR, 0.0–0.0). Most patients were admitted in the acute (70.97%) or subacute (22.58%) stage (Table 1).

The patients were grouped according to their mRS. There were no significant differences in clinical characteristics between the two groups (all *p* > 0.05) (Table 2).

**Table 2:** Characteristics of the patients according to the mRS.

	mRS > 0	mRS = 0	*p*
	(*n* = 6)	(*n* = 25)	
Sex			0.349
Male	1 (16.67%)	12 (48.00%)	
Female	5 (83.33%)	13 (52.00%)	
Age (years)	42.50 ± 18.75	41.80 ± 14.10	0.919
Hypertension	1 (16.67%)	2 (8.00%)	0.488^*^
History of stroke	0	1 (4.00%)	>0.999^*^
Thrombotic disorders	2 (33.33%)	2 (8.00%)	0.159^*^
History of pregnant	1 (16.67%)	5 (20.00%)	>0.999
History of delivery	1 (16.67%)	2 (8.00%)	0.488^*^
Smoking	1 (16.67%)	3 (12.00%)	>0.999^*^
Alcohol drinking	0	4 (16.00%)	0.561^*^
Stage			>0.999^*^
Acute	5 (83.33%)	17 (68.00%)	
Subacute	1 (16.67%)	6 (24.00%)	
Chronic	0	2 (8.00%)	
Symptoms at onset			
Headaches	4 (66.67%)	18 (72.00%)	>0.999
Limb numbness	1 (16.67%)	5 (20.00%)	>0.999
Speech impairment	2 (33.33%)	2 (8.00%)	0.159^*^
Consciousness disturbances	1 (16.67%)	1 (4.00%)	0.355^*^
Limb tremors/convulsions	0	2 (8.00%)	>0.999^*^
Vomiting	0	2 (8.00%)	>0.999^*^
Memory impairment	1 (16.67%)	1 (4.00%)	0.355^*^
Seizures	0	2 (8.00%)	>0.999^*^
Visual abnormalities	0	1 (4.00%)	>0.999^*^
Anticoagulation treatment			0.586^*^
300,000 U of urokinase in venous sinus in the morning and evening for 3 days	5 (83.33%)	19 (76.00%)	
500,000 U of urokinase in venous sinus once	0	1 (4.00%)	
Balloon dilatation	1 (16.67%)	1 (4.00%)	
Warfarin 3 mg orally once daily for 3 months	0	4 (16.00%)	
Hemorrhagic transformation	0	1 (4.00%)	>0.999^*^
Drainage obstruction	6 (100.00%)	22 (88.00%)	>0.999^*^
Venous recanalization	6 (100.00%)	24 (96.00%)	>0.999^*^
Venous involvement	0	3 (12.00%)	>0.999^*^
Intracranial pressure (mmH_2_O)	250 (227.5, 290)	220 (200, 315)	0.339
Onset to admission time (days)	1.92 (0.58, 5.50)	5 (3.25, 10.00)	0.174
NIHSS at admission			0.159^*^
0	3 (50.00)	15 (60.00)	
1–4	0	6 (24.00)	
≥ 5	3 (50.00)	4 (16.00)	

* Fisher’s exact test.

mRS, modified Rankin scale.

### 3.2 Treatments

The patients were treated with low-molecular-weight heparin (*n* = 6), interventional therapy (*n* = 2), warfarin (*n* = 7), low-molecular-weight heparin and interventional therapy (*n* = 6), low-molecular-weight heparin and warfarin (*n* = 1), and warfarin with interventional therapy (*n* = 9). Table 3 presents the characteristics of the patients according to treatment.

**Table 3:** Factors influencing treatment measures.

	Low-molecular-weight heparin (*n* = 6)	Interventional therapy (*n* = 2)	Warfarin (*n* = 7)	Low-molecular-weight heparin + interventional therapy (*n* = 6)	Low-molecular-weight heparin +warfarin (*n* = 1)	Warfarin+ interventional therapy (*n* = 9)	Overall (*n* = 31)
Sex							
Male	3 (50.00%)	0	4 (57.14%)	1 (16.67%)	1 (100.00%)	4 (44.44%)	13 (41.94%)
Female	3 (50.00%)	2 (100.00%)	3 (42.86%)	5 (83.33%)	0	5 (55.56%)	18 (58.06%)
Age (years)	50.5 [37.0, 55.0]	40.0 [38.0, 42.0]	38.0 [30.0, 67.0]	28.0 [13.0, 64.0]	48.0 [48.0, 48.0]	39.0 [23.0, 60.0]	39 (30, 53)
Hypertension	1 (16.67%)	0	0	2 (33.33%)	0	0	3 (9.68%)
Diabetes	0	0	0	0	0	0	0
CAD	0	0	0	0	0	0	0
Hyperlipidemia	0	0	0	0	0	0	0
History of stroke	0	0	1 (14.29%)	0	0	0	1 (3.23%)
Thrombotic disorders	0	0	0	1 (16.67)	1 (100.00%)	2 (22.22%)	4 (12.90%)
History of pregnant	0	1 (50.00%)	2 (28.57%)	0	0	3 (33.33%)	6 (19.35%)
History of delivery	0	0	1 (14.29%)	1 (16.67%)	0	1 (11.11%)	3 (9.68%)
Smoking	0	1 (50.00%)	2 (28.57%)	0	0	1 (11.11%)	4 (12.90%)
Alcohol drinking	1 (16.67%)	1 (50.00%)	1 (14.29%)	0	0	1 (11.11%)	4 (12.90%)
Stage							
Acute	4 (66.67%)	1 (50.00%)	4 (57.14%)	5 (83.33%)	1 (100.00%)	7 (77.78%)	22 (70.97%)
Subacute	1 (16.67%)	1 (50.00%)	2 (28.57%)	1 (16.67%)	0	2 (22.22%)	7 (22.58%)
Chronic	1 (16.67%)	0	1 (14.29%)	0	0	0	2 (6.45%)
Symptoms at onset							
Headaches	2 (33.33%)	2 (100.00%)	4 (57.14%)	4 (66.67%)	1 (100.00%)	9 (100.00%)	22 (70.97%)
Limb numbness	1 (16.67%)	1 (50.00%)	2 (28.57%)	0	0	2 (22.22%)	6 (19.35%)
Speech impairment	2 (33.33%)	0	0	0	1 (100.00%)	1 (11.11%)	4 (12.90%)
Consciousness disturbances	0	0	0	2 (33.33%)	0	0	2 (6.45%)
Limb tremors/convulsions	0	0	2 (28.57%)	0	0	0	2 (6.45%)
Vomiting	0	0	0	1 (16.67%)	0	1 (11.11%)	2 (6.45%)
Memory impairment	0	0	1 (14.29%)	1 (16.67%)	0	0	2 (6.45%)
Seizures	0	0	0	0	0	2 (22.22%)	2 (6.45%)
Visual abnormalities	1 (16.67%)	0	0	0	0	0	1 (3.23%)
Radiological location							
Straight sinus	1 (16.67%)	1 (50.00%)	2 (28.57%)	1 (16.67%)	1 (100.00%)	4 (44.44%)	10 (32.26%)
Transverse sinus	5 (83.33%)	1 (50.00%)	5 (71.43%)	4 (66.67%)	1 (100.00%)	6 (66.67%)	22 (70.97%)
Side							
Not specified	2 (33.33%)	0	0	0	0	1 (11.11%)	3 (13.64%)
Left	3 (50.00%)	1 (50.00%)	2 (28.57%)	2 (33.33%)	0	1 (11.11%)	9 (40.91%)
Right	0	0	3 (42.86%)	1 (16.67%)	1 (100.00%)	4 (44.44%)	9 (40.91%)
Bilateral	0	0	0	1 (16.67%)	0	0	1 (4.55%)
Sigmoid sinus	4 (66.67%)	0	1 (14.29%)	3 (50.00%)	0	6 (66.67%)	14 (45.16%)
Side							
Not specified	2 (33.33%)	0	1 (14.29%)	3 (50.00%)	0	4 (44.44%)	10 (71.43%)
Left	1 (16.67%)	0	0	0	0	0	1 (7.14%)
Right	1 (16.67%)	0	0	0	0	1 (11.11%)	2 (14.29%)
Bilateral	0	0	0	0	0	1 (11.11%)	1 (7.14%)
Superior sagittal sinus	1 (16.67%)	1 (50.00%)	4 (57.14%)	2 (33.33%)	0	7 (77.78%)	15 (48.39%)
Side							
Left	1 (16.67%)	1 (50.00%)	4 (57.14%)	2 (33.33%)	0	7 (77.78%)	15 (100.00%)
Internal jugular vein	0	0	0	1 (16.67%)	0	1 (11.11%)	2 (6.45%)
Side							
Not specified	0	0	0	0	0	1 (11.11%)	1 (50.00%)
Left	0	0	0	1 (16.67%)	0	0	1 (50.00%)
Cortical veins	0	0	1 (14.29%)	0	0	0	1 (3.23%)
MRV was performed	4 (66.67%)	2 (100.00%)	4 (57.14%)	6 (100.00%)	0	9 (100.00%)	25 (80.65%)
DSA was performed	4 (66.67%)	2 (100.00%)	5 (71.43%)	6 (100.00%)	1 (100.00%)	9 (100.00%)	27 (87.10%)
GCS at admission	15 (15, 15)	15 (15, 15)	15 (15, 15)	14.5 (13.75, 15)	–	15 (13.5, 15)	15 (15, 15)
NIHSS at admission							
0	3 (50.00%)	0	5 (71.43%)	4 (66.67%)	1 (100.00%)	5 (55.56%)	18 (58.06%)
1–4	2 (33.33%)	1 (50.00%)	2 (28.57%)	1 (16.67%)	0	0	6 (19.35%)
≥ 5	1 (16.67)	1 (50.00%)	0	1 (16.67%)	0	4 (44.44%)	7 (22.58%)
mRS at admission							
> 0	1 (16.67%)	1 (50.00%)	0	3 (50.00%)	0	1 (11.11%)	6 (19.4%)
0	5 (83.33%)	1 (50.00%)	7 (100.00%)	3 (50.00%)	1 (100.00%)	8 (88.89%)	25 (80.6%)
Anticoagulation treatment							
300,000 U of urokinase in venous sinus in the morning and evening for 3 days	4 (66.67%)	2 (100.00%)	4 (57.14%)	5 (83.33)	1 (100.00%)	8 (88.89%)	24 (77.42%)
500,000 U of urokinase in venous sinus once	0	0	0	0	0	1 (11.11%)	1 (3.23%)
Balloon dilatation	0	0	1 (14.29%)	1 (16.67%)	0	0	2 (6.45%)
Warfarin 3 mg orally once daily for 3 months	2 (33.33%)	0	2 (28.57%)	0	0	0	4 (12.90%)
Hemorrhagic transformation	0	0	1 (14.29%)	0	0	0	1 (3.23%)
Drainage obstruction	4 (66.67%)	2 (100.00%)	6 (85.71%)	6 (100.00%)	1 (100.00%)	9 (100.00%)	28 (90.32%)
Venous recanalization	6 (100.00%)	2 (100.00%)	6 (85.71%)	6 (100.00%)	1 (100.00%)	9 (100.00%)	30 (96.77%)
Intracranial pressure (mmH_2_O)	220 (202.5, 245)	320 (260, 380)	210 (200, 300)	255 (227.5, 312.5)	–	220 (207.5, 335)	230 (210, 310)
Onset to admission time	6.00 (4.00, 45.00)	10.92 (0.83, 21.00)	6.00 (0.29, 20.00)	3.00 (0.18, 5.50)	–	5.00 (3.50, 8.50)	5 (3, 10)

MRV, magnetic resonance venography; DSA, digital subtraction angiography; GCS, Glasgow coma scale; NIHSS, National Institutes of Health stroke scale; mRS, modified Rankin scale.

### 3.3 Prognosis

**Figure 1:**
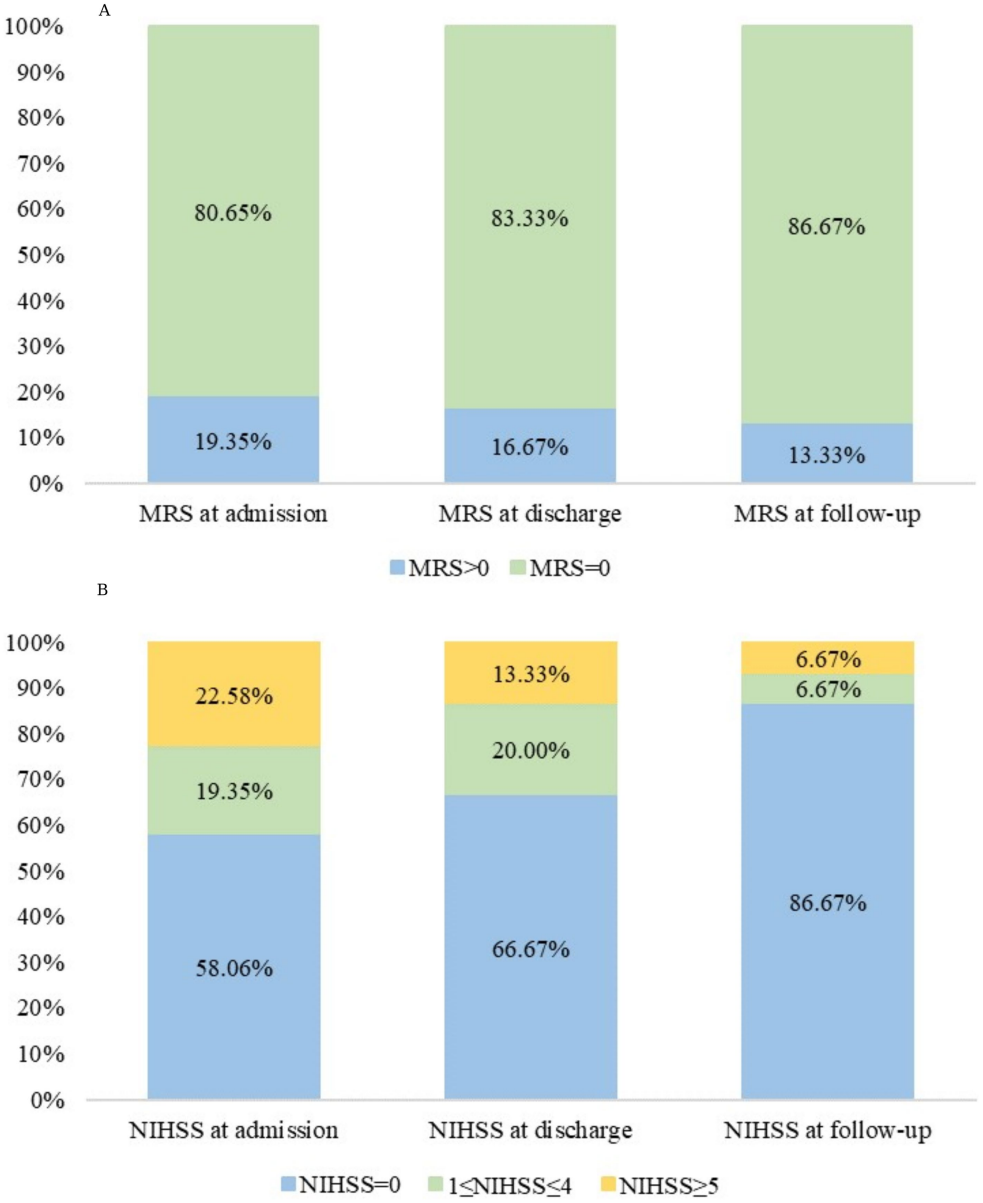
(A) Modified Rankin Scale (mRS) and (B) National Institutes of Health Stroke Scale (NIHSS) scores at admission, at discharge, and at the 3-month follow-up in patients with cerebral venous sinus thrombosis (CVST).

Hemorrhagic transformation was observed in one patient (3.23%), drainage obstruction in 28 (90.32%), and venous recanalization in 30 (96.77%). The intracranial pressure was 230 (IQR, 210–310) mmH_2_O (Table 1). Table 4 presents the characteristics of the patients according to the mRS at 3 months (>0 vs. 0). No factors were significantly associated with the mRS at 3 months (all *p* > 0.05), except for an NIHSS of >0 at follow-up that was associated with a 3-month mRS >0 (*p* < 0.001). Figure 1 shows the changes in mRS and NIHSS at admission, discharge, and 3-month follow-up.

**Table 4:** Outcome variable based on mRS score at 3 months.

	mRS > 0	mRS = 0	*p*
	*n* = 4	*n* = 26	
Sex			0.228
Male	0	12 (46.15%)	
Female	4 (100%)	14 (53.85%)	
Age (years)	45.75 ± 15.92	40.42 ± 14.24	0.498
Hypertension	1 (25.00%)	2 (7.69%)	0.360^*^
History of stroke	0	1 (3.85%)	>0.999^*^
Thrombotic disorders	0	4 (15.38%)	>0.999^*^
History of pregnant	1 (25.00%)	5 (19.23%)	>0.999^*^
History of delivery	1 (25.00%)	2 (7.69%)	0.360^*^
Smoking	1 (25.00%)	3 (11.54%)	0.454^*^
Alcohol drinking	1 (25.00%)	3 (11.54%)	0.454^*^
Stage			0.396^*^
Acute	2 (50.00%)	20 (76.92%)	
Subacute	2 (50.00%)	4 (15.38%)	
Chronic	0	2 (7.69%)	
Symptoms at onset			
Headaches	2 (50.00%)	20 (76.92%)	0.599
Limb numbness	1 (25.00%)	5 (19.23%)	>0.999^*^
Speech impairment	1 (25.00%)	3 (11.54%)	0.454^*^
Consciousness disturbances	0	2 (7.69%)	>0.999^*^
Limb tremors/convulsions	0	2 (7.69%)	>0.999^*^
Vomiting	0	2 (7.69%)	>0.999^*^
Memory impairment	1 (25.00%)	0	0.133^*^
Seizures	1 (25.00%)	1 (3.85%)	0.253^*^
Visual abnormalities	0	1 (3.85%)	>0.999^*^
Radiological location			
Straight sinus	1 (25.00%)	8 (30.77%)	>0.999
Transverse sinus	3 (75.00%)	18 (69.23%)	>0.999
Side			0.783^*^
Not specified	0	3 (16.67%)	
Left	2 (66.67%)	6 (33.33%)	
Right	1 (33.33%)	8 (44.44%)	
Bilateral	0	1 (5.56%)	
Sigmoid sinus	1 (25.00%)	13 (50.00%)	0.693
Side			>0.999^*^
Not specified	1 (100.00%)	9 (69.23%)	
Left	0	1 (7.69%)	
Right	0	2 (15.38%)	
Bilateral	0	1 (7.69%)	
Superior sagittal sinus	2 (50.00%)	13 (50.00%)	>0.999
Side			
Left	2 (100.00%)	13 (100%)	–
Internal jugular vein	0	2 (7.69%)	>0.999^*^
Side			–
Not specified	0	1 (50.00%)	
Left	0	1 (50.00%)	
Cortical veins	0	1 (3.85%)	>0.999^*^
MRV was performed	3 (75.00%)	21 (80.77%)	>0.999^*^
DSA was performed	4 (100.00%)	22 (84.62%)	>0.999^*^
Anticoagulation treatment			>0.999^*^
300,000 U of urokinase in venous sinus in the morning and evening for 3 days	4 (100.00%)	19 (73.08%)	
500,000 U of urokinase in venous sinus once	0	1 (3.85%)	
Balloon dilatation	0	2 (7.69%)	
Warfarin 3 mg orally once daily for 3 months	0	4 (15.38%)	
Hemorrhagic transformation	0	0	–
Drainage obstruction	4 (100.00%)	23 (88.46%)	>0.999^*^
Venous recanalization	4 (100.00%)	26 (100.00%)	–
Venous involvement	0	3 (11.54%)	–
Intracranial pressure (mmH_2_O)	330 (260, 370)	220 (200, 265)	0.009
Onset to admission time	8.50 (4.75, 18.25)	4.50 (2.46, 7.75)	0.177
NIHSS at follow-up			<0.001^*^
0	0	26 (100.00%)	
1–4	2 (50.00%)	0	
≥5	2 (50.00%)	0	

MRV, magnetic resonance venography; DSA, digital subtraction angiography.

* Fisher’s exact test.

## 4 Discussion

CVST is a rare condition. Its epidemiology is poorly known, especially with the improvements in diagnostic modalities and the diagnosis of mild cases. Therefore, this retrospective study examined the etiology, treatment response, and prognosis of patients with CVST at a single institution. The median age was 39 years. The majority were female (58%), 13% had thrombotic disorders, and 19% were or were recently pregnant. Only an NIHSS of >0 at follow-up was associated with a 3-month mRS >0.

This case series was small, but the results were consistent with those previously reported. Indeed, Khan et al. (6) reported 33 patients from Pakistan; 88% of their patients were female, and the mean age at presentation was 31 years. Ibrahim et al. (15) reported a median age of presentation of 35 (range, 23–75) years. Similar results were reported by Bousser et al. (16) and in a systematic review by Coutinho et al. (9). In China, Qiu et al. (17) reported that females were predominant and that the age at presentation was 20–40 years, also supporting the present study. A multicenter study of 11,400 patients with CVST reported a mean age at presentation of 38.1 years (18).

In the present study, 19% of the patients had an mRS >0 at presentation, while previous studies reported 32–39% of patients with altered consciousness (6, 19). The discrepancies could be due to differences in sample size, patient populations, and diagnostic methods. Headaches were the most common symptom. Although headaches are non-specific, they are the most common symptoms reported in the CVST literature, while other neurological symptoms were reported in 30–50% of the patients (6, 15, 19–22).

An international study revealed that the most common sinus involvement was the superior sagittal sinus (62%), followed by the transverse sinus (42%), straight sinus (18%), cortical veins (17%), and deep veins (11%) (23). Khan et al. (6) showed that the transverse sinus was commonly involved (55%), followed by the sigmoid sinus (52%), superior sagittal sinus (46%), and straight sinus (15%), similar to Bousser et al. (16) and Banakar et al. (24). In the present study, the transverse sinus (71.0%), superior sagittal sinus (48.4%), sigmoid sinus (45.2%), straight sinus (32.3%), internal jugular vein (6.45%), and cortical veins (3.23%) were involved. Discrepancies among studies could be due to differences in scanners, imaging modalities and parameters, and radiologist experience. Of note, magnetic resonance has a sensitivity of 72–84% and a specificity of 89.9–95% for CVST (25, 26).

The most important feature of CVST management is early recognition and anticoagulation (2, 21). Concomitant conditions like infections and seizures should also be managed appropriately. Other conditions should also be managed (e.g., edema, dehydration, and malnutrition).

In the present study, 86.7% of the patients had an mRS of 0 at 3 months, indicating excellent outcomes. No deaths were observed. Previous studies reported good outcomes in 79% of the patients, in-hospital mortality of 2.0%, mortality of 9–15%, and 1-year recurrence of 6.5% (18, 20, 21). A previous study showed that headaches were a protective manifestation of CVST, while consciousness disturbances, intracranial injury, and hematologic diseases indicated poor clinical prognosis; no significant associations were found between the number and location of lesions and clinical prognosis (22). Factors of poor outcomes also include age > 33–37 years, male sex, coma, neurologic deficit on NIHSS, encephalopathy, decreased level of consciousness, hemiparesis, seizures, intracerebral hemorrhage, involvement of straight sinus, thrombosis of deep vein system, venous infarction, cancer, central nervous system infection, and underlying coagulopathy (1, 8). Khan et al. (6) reported that the factors of poor prognosis in women with CVST were spontaneous vaginal delivery, primigravida, and anemia, but most of their patients were postpartum. Additional studies are still necessary to determine poor prognosis factors in patients with CVST. In the present study, an NIHSS of >0 at follow-up was associated with a 3-month mRS >0. The NIHSS was designed to quantify the impairments after a stroke (27, 28). The mRS measures the degree of disability or dependence on daily activities after a stroke or other neurological disabilities (29). Therefore, although there are differences between the two scales, they examine a similar outcome, and their association is not surprising.

This study had limitations. It was a single-center study spanning a relatively short period, resulting in a small sample size. The study was retrospective, limiting the data to those available in the patient charts. The 3-month outcomes were available for 30 patients. Long-term outcomes were unavailable for most patients since their mRS at 3 months was good, and many patients were returned to their local hospital. A risk score model could not be developed because of the small sample size. A multicenter study would increase the sample size and probably allow the development of a risk score model. The treatment groups were too small to allow statistical analyses and investigate the differences in patient characteristics and outcomes among the treatment groups. Therefore, only a description of the factors was possible. A multicenter study would allow the investigation of the effect of different treatments on patient outcomes and the clinical factors that could lead to the selection of a treatment pattern.

In conclusion, this case series reported the features of patients with CVST treated at a single institution in recent years. Although all patients with CVST diagnosed during the study period were included, most had mild CVST. In addition, the majority of patients were female. The median age was 39 years, 13% had thrombotic disorders, and 19% had a pregnancy history. Only an NIHSS of >0 at follow-up was associated with a 3-month mRS >0. Multicenter studies should be performed to examine the prognostic factors of CVST in a larger patient population.

## Data Availability

The original contributions presented in the study are included in the article/supplementary material, further inquiries can be directed to the corresponding author.
